# Preferences for Genetic Testing to Predict the Risk of Developing Hereditary Cancer: A Systematic Review of Discrete Choice Experiments

**DOI:** 10.1177/0272989X241227425

**Published:** 2024-02-07

**Authors:** N. Morrish, T. Snowsill, S. Dodman, A. Medina-Lara

**Affiliations:** Public Health Economics Group, Department of Public Health and Sport Sciences, Faculty of Health and Life Sciences, University of Exeter, Exeter, UK; Health Economics Group, Health and Community Sciences, Faculty of Health and Life Sciences, University of Exeter, Exeter, UK; University of Exeter, Exeter, UK; Public Health Economics Group, Department of Public Health and Sport Sciences, Faculty of Health and Life Sciences, University of Exeter, Exeter, UK

**Keywords:** genetic testing, hereditary cancer, systematic review, discrete choice experiment

## Abstract

**Background:**

Understanding service user preferences is key to effective health care decision making and efficient resource allocation. It is of particular importance in the management of high-risk patients in whom predictive genetic testing can alter health outcomes.

**Purpose:**

This review aims to identify the relative importance and willingness to pay for attributes of genetic testing in hereditary cancer syndromes.

**Data Sources:**

Searches were conducted in Medline, Embase, PsycINFO, HMIC, Web of Science, and EconLit using discrete choice experiment (DCE) terms combined with terms related to hereditary cancer syndromes, malignancy synonyms, and genetic testing.

**Study Selection:**

Following independent screening by 3 reviewers, 7 studies fulfilled the inclusion criteria, being a DCE investigating patient or public preferences related to predictive genetic testing for hereditary cancer syndromes.

**Data Extraction:**

Extracted data included study and respondent characteristics, DCE attributes and levels, methods of data analysis and interpretation, and key study findings.

**Data Synthesis:**

Studies covered colorectal, breast, and ovarian cancer syndromes. Results were summarized in a narrative synthesis and the quality assessed using the Lancsar and Louviere framework.

**Limitations:**

This review focuses only on DCE design and testing for hereditary cancer syndromes rather than other complex diseases. Challenges also arose from heterogeneity in attributes and levels.

**Conclusions:**

Test effectiveness and detection rates were consistently important to respondents and thus should be prioritized by policy makers. Accuracy, cost, and wait time, while also important, showed variation between studies, although overall reduction in cost may improve uptake. Patients and the public would be willing to pay for improved detection and clinician over insurance provider involvement. Future studies should seek to contextualize findings by considering the impact of sociodemographic characteristics, health system coverage, and insurance policies on preferences.

**Highlights:**

- Test effectiveness and detection rates are consistently important to respondents in genetic testing for hereditary cancer syndromes.
- Reducing the cost of genetic testing for hereditary cancer syndromes may improve uptake.
- Individuals are most willing to pay for a test that improves detection rates, identifies multiple cancers, and for which results are shared with a doctor rather than with an insurance provider.

## Introduction

Efficient allocation of resources is fundamental to the success and sustainability of health services. Concurrently, there is increasing recognition of the value in incorporating public (taxpayers or insurance premium payers) and patient (care recipient) preferences into health care decision making.^1^ Including these preferences in the design and configuration of health services could maximize utility, patient satisfaction, adherence, and uptake while simultaneously improving the efficiency of services.^2,3^

Hereditary cancer is caused by the inheritance of genetic variants that confer a higher risk of developing malignancies.^4^ High-penetrance alleles have been implicated in the pathogenesis of several hereditary cancer syndromes including hereditary breast and ovarian cancer,^5,6^ Lynch syndrome,^7^ and familial adenomatous polyposis.^8^ For individuals with a family history suggestive of a hereditary cancer syndrome, testing for high-risk alleles can guide treatment management decisions such as screening frequency or risk-reducing surgery decisions.^9,10^ The decision to undergo genetic cancer testing comes with several challenges, including frequent co-occurrence with a recent cancer diagnosis, resulting implications for family members, and implications for childbearing. Understanding public and patient preferences is therefore of particular importance given the complexity of the decision-making process.

The consensus is that screening at-risk individuals for high-penetrance alleles associated with hereditary cancer syndromes improves health outcomes, can increase the opportunity for testing in wider family members, and is perceived as a relevant and useful clinical intervention. Nonetheless, the estimated uptake of predictive genetic testing in populations at risk for a hereditary cancer syndrome is only 50%.^11^ Reasons cited for opting out of testing in high-risk populations include lack of knowledge regarding test availability, distrust of test results, belief that testing is unlikely to alter health outcomes, sense that the negative effects of testing will outweigh the potential benefits, and concerns regarding insurance discrimination.^12,13^

Existing research highlights the importance of ensuring all at-risk individuals are offered testing and of informing eligible candidates of the potential testing benefits to facilitate informed decision making. It is widely recognized that clinical outcomes affect medical decision making; however, the impacts of process attributes, such as test characteristics and service characteristics, are less well understood.^14^ Understanding individuals’ preferences for relevant predictive genetic testing attributes is vital to stakeholders and service providers to maximize patient and service utility.

Discrete choice experiments (DCEs) are increasingly used in health service research to understand public and patient preferences for health care interventions and in some cases test characteristics (attributes).^15,16^ In a DCE, respondent preferences are obtained through a series of hypothetical choice sets, each describing an intervention, service, or test with varying attribute levels.^17^ Respondents’ choices are then analyzed to understand the tradeoffs and participant preferences for the intervention, service, or test attributes assessed.^17^ DCE findings can be used to predict the probability of uptake and (if cost is included as an attribute) provide an indication of willingness to pay (WTP) for services.^17^

This review adds to the literature and extends recent work by Ozdemir et al.^18^ through focused consideration of genetic testing in cancer. It aims to identify the factors affecting the decision to undergo genetic testing for hereditary cancer syndromes by reviewing relevant DCEs, comparing attributes and levels used in each study, and ultimately investigating patient and public preferences for predictive testing for hereditary cancer.

## Methods

### Identification of Studies

Information specialists searched to August 31, 2022, in Medline, Embase, PsycINFO, HMIC, Web of Science, and EconLit using pretested published terms for discrete choice studies.^15^ DCE hits were combined into a single database in EndNote X9, which was searched using terms related to named adult hereditary cancer syndromes, general terms for malignancy, and genetic testing, as detailed in Appendix A. Forward and backward citation chasing was conducted on included studies.

### Study Selection

Titles and abstracts were independently screened by 2 reviewers (from a selection of S.D., N.M., T.S.) to identify DCEs investigating public or patient preferences related to genetic testing in oncology. This included genetic testing for the purposes of risk prediction, pharmacogenetics, and diagnosis. Studies meeting these initial inclusion criteria were reviewed at full text by 2 of the 3 available reviewers (S.D., N.M., T.S.) using the criteria below.

DCEs included at full text were to

- investigate patient or public preferences related to genetic testing for hereditary cancer syndromes;
- investigate genetic or genomic testing of constitutional DNA to determine whether the patient is at higher than average risk for developing cancer due to genetic variation;
- relate to a single gene or polygenic risk score, provided the genes were hereditary; and
- consider predictive genetic testing (currently unaffected individuals) or diagnostic genetic testing (people with preexisting cancer).

Exclusion criteria were the following:

- genome testing for personalized cancer treatment
- conjoint analyses (excluded at both screening stages), and
- non–English language studies.

Results were summarized in line with PRISMA guidelines.^19^

### Data Extraction and Quality Appraisal

Data were extracted on study characteristics (population, cancer type, survey administration), respondent characteristics (age, gender, income, education), DCE attributes and levels, methods of data analysis and interpretation, and key study findings. Reporting quality was assessed using a quality appraisal checklist published by Lancsar and Louviere.^17^ This checklist does not include a scoring system but considers the transparency of reporting regarding each stage of the DCE without including judgments on whether particular methods improve or worsen validity. Data extraction and quality appraisal were conducted by 1 of 2 reviewers (S.D., N.M.), with all studies checked by a third reviewer (A.M.L.).

### Analysis and Synthesis

In each study, the attribute with the greatest impact on test utility was assigned a preference weight of 1, and all other attributes were scaled relative to this attribute by dividing their relative importance score by that of the most preferred attribute, to facilitate cross-study comparison. WTP and test uptake costs were converted to 2022 US$ using International Monetary Fund (IMF) purchasing power parity values. Prices were inflated from the year of data collection or, where not reported, inflated from the year prior to publication. Results are summarized in a narrative synthesis supported by cross-tabulation.

## Results

**Figure 1:**
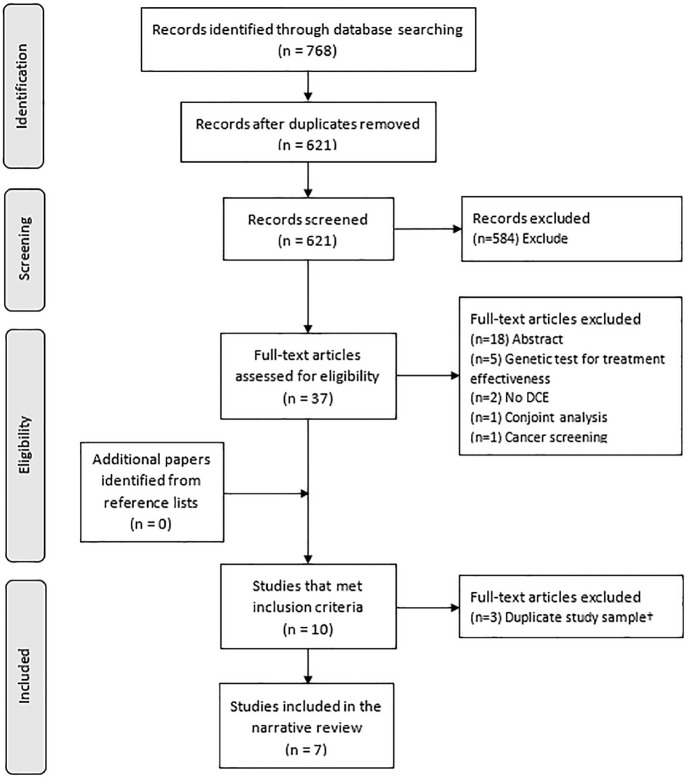
PRISMA diagram. From Moher et al.^19^ Available from http://journals.plos.org/plosmedicine/article?id=10.1371/journal.pmed.1000097. ^†^The 10 studies that met the inclusion criteria for this review described the results of 7 different discrete choice experiments; therefore, 3 studies were excluded from the narrative review, at full-text screening, to prevent double counting of data.

Electronic database searches identified 768 studies, of which 621 were unique records following deduplication. Of these, 584 were excluded during title and abstract screening and 27 excluded at full-text review. The 10 studies included at full text reported the results of 7 unique discrete choice studies. Three studies^20–22^ were excluded to prevent double counting of data as they used the same study sample and attributes as other included studies, leaving 7 studies summarized in a narrative synthesis. Further details are presented in the PRISMA diagram (Figure 1).

### Study Characteristics

The earliest included study was published in 2009,^23^ while all other studies have been published more recently, from 2015 onward. Three studies were conducted in the United States^24–26^ and 1 each in the United Kingdom,^23^ the Netherlands,^27^ Singapore,^28^ and Australia.^29^ Two studies recruited patient populations at risk for a Hereditary Cancer Syndrome,^23,24^ 1 recruited patients with a diagnosis of epithelial ovarian cancer,^26^ and 4 recruited representative samples of the general population.^25,27–29^ Most studies considered a single hereditary cancer: colorectal,^24,25,27^ breast,^28^ or ovarian.^26^ One study considered polygenic risk score,^29^ and 1 study evaluated the risk of developing genetic breast, ovarian, or colorectal cancer.^23^ DCEs were predominantly administered online.^24,25,27,29^ One study opted for a mailed questionnaire,^23^ 1 conducted a face-to-face survey,^28^ and 1 did not report the mode of administration.^26^ More details are described in Table 1.

**Table 1:** Study Characteristics

	Griffith 2009	Knight 2015	Veldwijk 2016	Weymann 2018	Wong 2018	Davidson 2019	Venning 2022
Country	Wales	United States	Netherlands	United States	Singapore	United States	Australia
Study aim	Demand, WTP, and patient preferences for genetic testing and cancer genetic services	Relative value of specific characteristics of genetic tests	Test whether framing a risk attribute positively or negatively influences decision making-behavior and preferences	Monetary value and preference-based utility of MPS sequencing and testing for CRC risk	Marginal WTP for the single-nucleotide polymorphism gene test	Preferences for tailoring genetic counseling and test selection	Polygenic risk score test attributes most influence the likelihood of testing
Population	Patients at high, moderate, and low risk of developing genetic cancer (breast, ovarian, and colorectal)	Probability based sample of US adults aged 50+ y	Representative sample of the Dutch population aged 55–65 y	Patients with a personal and/or family history of colon cancer and/or polyposis or other features of Lynch syndrome	English-speaking Singaporean women aged 40–69 y without history of breast cancer	English-speaking women aged 18+ y with a diagnosis of EOC referred for germline genetic testing	Representative sample of Australian participants aged 18+ y
Cancer type	Breast, ovarian, and colorectal	Colorectal	Colorectal	Colorectal	Breast	Ovarian	Polygenic risk score
Mode of administration	Mailed questionnaires	Online survey	Online survey	Online survey	Face-to-face survey	Not reported	Online survey

CRC, colorectal cancer; EOC, epithelial ovarian cancer; MPS, massively parallel sequencing; NEXT, new exome technology; WTP, willingness to pay.

#### DCE characteristics

Studies included between 4 and 8 attributes, with a minimum of 2 and maximum of 6 levels. These attributes covered service design, cost, and results/impact. Further detail of individual attributes and levels is provided in Table 2. Respondents were presented with between nine^25,27^ and twenty-three^23^ choice sets. Four studies used a blocked design,^24–26,29^ of which 1 randomly assigned respondents to 1 of 4 versions of the survey.^25^ All DCEs were designed using a fractional factorial design with main effects. Two studies included interactions alongside main effects.^28,29^ One study used dummy coding of attributes,^29^ while all others effects-coded attributes (n = 6), of which 2 also included continuous coding for a selection of attributes.^24,28^ Data were analyzed using mixed-logit specification (n = 6), 1 of which also used latent class analysis^29^ or multinomial logit regression (n = 1).^23^ Mixed logit specification relaxes the assumption of independent errors, allowing unrestricted patterns of substitution and random parameters account for preference heterogeneity across individuals.^30^ The random parameters approach is particularly appropriate where data show considerable preference heterogeneity; however, it is limited by the need for a larger sample size, which was not necessarily achieved by the included studies.

**Table 2:** DCE Characteristics

	Griffith 2009	Knight 2015	Veldwijk 2016	Weymann 2018	Wong 2018	Davidson 2019	Venning 2022
Number of attributes included	6	4	4	4	4	5	8 (to reduce complexity, used partial profiles with 2 overlapping attributes)
Attributes and levels (mid gray = cost; light gray = service design; dark gray = results and impact)	Cost of testing service •£1,500 •£2,000 •£2,500^a^ •£3,000	Personal cost not covered by insurance • $250 • $500 • $1,000 or $1,500^b^	Colonoscopy frequency • 1 y • 2 y • 5 y	Total cost to you • $425 • $1,000 • $1,900 • $2,550	Out-of-pocket cost • S$50 (US$38) • S$175 (US$134) • S$300 (US$229)	Out-of-pocket cost • $ 0 • $200 • $1,000 • $5,000	Test cost • $ 0 (no cost) • $75 ($53 US) • $150 ($107 US)
	Duration of counseling • 30 min • 1 h^a^ • 1 h 30 min • 2 h	Chance of a false-negative test result • 0% • 10% • 20%	Genetic predisposition to CRC • 1% • 3% • 15%	Number of genetic tests required • 1 • 2 • 4 • 5	Sample type • Buccal swab • Dried blood spot	Collection • Saliva • Blood	To have the test and receive the results you would • Order test online, perform cheek swab at home, send swab back, and receive results online • Go to GP, have cheek swab taken, see GP for the results • Obtain referral to genetic specialist, have cheek swab taken, see genetic specialist for results
	Distance to counseling • 20 miles • 40 miles^a^ • 60 miles • 80 miles	Chance of getting CRC • 10% • 25% • 50%	Probability of developing CRC • 15% • 70% • 99%	Time waiting for genetic test results • 3 wk • 1.5 m • 3 mo • 6 mo	Test location • Private family clinic • Hospital • Public primary-care clinic	Turnaround time • 1 wk • 2 wk • 4 wk	Type of cancer the test for is • Pancreatic • Breast/prostate (reflexive based on gender) • Bowel • Melanoma • Lung • Pancreatic, breast, prostate, bowel, melanoma, and lung (multiple cancer test)
	Staff seen for counseling • Specialist genetics nurse^a^ • Consultant geneticist • Genetics associate	Who else sees the test results • Primary care doctor • Genetics health professionals • Life insurance and health insurance companies	Probability of surviving CRC^c^ • 80% • 92% • 98%	Tested individuals receiving a genetic diagnosis causing CRC • 40% • 60% • 80% • 90%	Person conducting pretest discussion • Specialist doctor • Nurse educator trained in genetic counseling • Nonspecialist doctor	Chance of finding a genetic mutation, if have one • 88% • 80% • 60%	Test accuracy for cancer risk • 60% • 75% • 90%
	Waiting time for letter confirming risk status • 1 mo • 2 mo^a^ • 4 mo • 6 mo		Probability of dying from CRC^c^ • 20% • 8% • 2%			Chance of finding an uncertain variant • 5% • 20% • 40%	Chances your recommended cancer screening changes as a result of the test • 10% • 25% • 50%
	Availability of genetics testing • Testing for high risk only • Testing for all^a^						If test indicates high risk, to help reduce your risk you can • There are no specific changes you can make to reduce your risk of this cancer • Participate in cancer screening • Make lifestyle changes • Participate in cancer screening and make lifestyle changes • Take a medication that reduces your cancer risk • Have surgery to reduce the chance the cancer will occur
							The result of the DNA test can affect whether you would qualify for life insurance or if your life insurance premiums would be affected • Yes • No
							Who has access to the result • Only me • Me and my family members • Me and my health professionals
Number of alternatives presented in each choice set	2+ indifference option	2+ opt-out	2+ opt-out choice afterward	2+ opt-out	2	2	2
Number of choice sets per respondents	23	9	9	16	10	10	12
Number of surveys generated	1	4	2 surveys with either positive (survival) or negative (mortality) attribute framing	2	1	21	36
Design type	Fractional factorial	Fractional factorial	Fractional factorial	Fractional factorial	Fractional factorial	Fractional factorial	Fractional factorial
Design properties	Main effects	Main effects	Main effects	Main effects	Main effects and interactions	Main effects	Main effects (also interactions for breast/prostate cancer)
Coding	Effects coded	Effects coded	Effects coded	Effects coded for individuals with variant detected; continuous otherwise	Out-of-pocket costs coded continuous; effects coded otherwise	Effects coded	Dummy coded
Method of data analysis	Multinomial logit regression	Mixed logit models	Panel mixed-logit model	Error-component mixed logit model	Mixed logit model	Mixed logit model	Mixed logit modelling and latent class analysis

CRC, colorectal cancer; GP, general practitioner.

a Attribute levels included in the constant comparator scenario.

b Half of the questionnaires said $1,000, half $1,500.

c Two versions of the survey were administered to respondents: in half of these surveys, the risk attribute was either positively framed (survival) or negatively framed (mortality).

#### Respondent characteristics

Respondent characteristics are presented in Table 3. The sample size ranged from 94 respondents^26^ to 1,045 respondents,^27^ although it clustered around either 100, 300, or 1,000 respondents. The response rate varied from 50.2%^28^ to 83%^29^ where reported. Participants were generally of older and middle age with mean age ranging from 45 y^23^ to 63 y.^25^ Studies contained either close to equal distribution of male and female respondents, or a fully female population. Six studies recorded educational attainment. In one study 90% of participants had some form of further education,^24^ while in another only 9% had college level training^28^ both of which are in contrast with the other studies which were more equally split across different academic levels.

**Table 3:** Respondent Characteristics

	Griffith 2009	Knight 2015	Veldwijk 2016	Weymann 2018	Wong 2018	Davidson 2019	Venning 2022
Response rate (%)	54.5	70	NR	66	50.2	NR	83^a^
Number of surveys completed	115^b^	451	1,262	122	300	103^c^	1,002^d^
Number of respondents included in the final analyses	115	355	1,045	122	300	94	1,002
Age, y	$\bar{x}$ : 44.53 *s*: 10.77	$\bar{x}$ : 63 Range: 50–96	$\bar{x}$ : 59.7 *s*: 3.1	Median: 55 IQR: 44–61	$\bar{x}$ : 52.6 *s*: 7.6	Median: 66 Range: 37–82	18+
Sex (% female)	98.3	50	49.7	53	100	100	50.3
Annual household income	NR	22% (<$25k) 28% ($25–50k) 23% ($50-75k) 13% ($75–100k) 14% (>$100k)	NR	10% (<$25k) 11% ($25–50k) 33% ($50–100k) 39% (>$100k) 7% (unknown)	29% (<S$36k) 54% (S$36k-84k) 16% (>S$84k)	NR	NR
Education	59% ≤GCSE 12% A level 23% ≥degree 6% other	11% <high school 34% high school 30% some college 24% ≥degree	26% low 37% medium 37% high	11% ≤high school 66% college/vocational 24% professional/graduate	16% ≤elementary 75% high school 9% ≥college	NR	27% school 28% vocational 44% university

GCSE, general certificate of secondary education; IQR, interquartile range; $\bar{x}$ , mean; NR, not reported; *s*, standard deviation; S$, Singapore dollar.

a Defined as the number of participants starting the survey divided by the number of online panel members invited to the survey.

b A total of 120 respondents returned the discrete choice questionnaire; however 5 of the questionnaires were incomplete.

c A total of 114 patients consented. Of these, 20 were excluded: 4 pilot subjects, 4 screen failures, 1 duplicate, and 11 did not complete the survey.

d The completion rate was 92% (total number of participants who completed the survey divided by total number who started the survey).

### Quality Appraisal

Quality appraisal focused on conceptualization, design, data collection, and analysis of the DCE as guided by the quality checklist.^17^ Further details of the quality appraisal can be found in Appendix B.

#### Conceptualizing the DCE

Six studies reported a literature review, clinical expert consultation, and interviews or focus groups with the target population or patient groups used to identify and select attributes. Despite this methodological similarity, the coverage of attributes varied widely across studies. The seventh study selected 4 attributes based on a single published study and reported that the additional 2 attributes, cost and availability of testing, were included to assess the aims of the study.^23^

All included studies presented generic alternatives within their choice tasks. Within each choice set, 3 studies included only the testing alternative,^26,28,29^ 1 presented 2 testing alternatives and an opt-out,^24^ 2 used forced-choice design followed by an opt-out option,^25,27^ and 1 offered an indifference choice between the pair of testing alternatives presented.^23^

DCE piloting was poorly reported across all studies and not reported at all in 2 cases.^23,24^ Where reported, it was used to check understanding and/or complexity. Only 2 studies reported the use of piloting to check coverage of the attributes and levels.^25,27^ No study explicitly reported how long it took to complete the DCE during piloting.

#### DCE design

Assessment of experimental design quality was limited due to poor reporting across studies. All included studies used fractional factorial designs and investigated main effects. Two also considered interaction terms.^28,29^ Only 1 study reported conducting sample size calculations (based on Orme’s Rule of Thumb).^28^ This study was also the only study to provide an incentive to enhance response rates. Three studies reported use of software packages to generate a D-efficient design,^24,25,27^ of which 2 used D-optimal algorithms to maximixe efficiency,^24,25^ however none of the studies reported the efficiency of the design. One study used an orthogonal array in which each choice set included a constant comparator profile that described current practice at the genetic testing service, which was compared against another alternative.^23^ Another used orthogonal array and partial profiles with overlapping attributes to improve complexity and efficiency.^29^ The remaining 2 studies were unclear in their reporting of design efficiency.^26,28^

#### Data collection

Three studies recruited patient populations at risk for a hereditary cancer syndrome based on personal and/or family history^23,24^ or a diagnosis of epithelial ovarian cancer.^26^ Four studies recruited participants from the general population necessitating hypothetical contemplation of risk status and testing. This was contextualized to participants through an explanation of gene testing prior to completing the DCE,^28^ description of a hypothetical scenario before presenting choice tasks,^29^ or incorporation of a hypothetical level of colorectal cancer risk as an attribute to provide a baseline context for each choice.^25,27^

#### Data analysis and interpretation

Four studies excluded respondent data that did not meet quality controls, for example those that completed the task at an unexpectedly rapid pace, consistently chose the same option, considered the DCE task difficult, or provided incomplete responses.^25–28^ Three studies effects coded^31^ all attributes (i.e., assuming the effects are uncorrelated with the intercept).^23,25–27^ One study effects coded only individuals with a genetic variant detected,^24^ and another effects coded all but the cost attribute.^28^ The final study dummy coded all attributes.^29^ One study reported that model specification was selected based on model fit tests.^27^ A further 2 studies calculated *R*^2^ statistics for goodness-of-fit and/or calculated Akaike and Bayesian information criteria; however, neither study used them to inform model specification.^23,29^ One study tested the internal validity of the DCE; however, in this study, responses that violated random utility theory axioms were not excluded.^23^

### Factors Affecting the Utility of Testing

#### Relative importance of attributes

**Figure 2:**
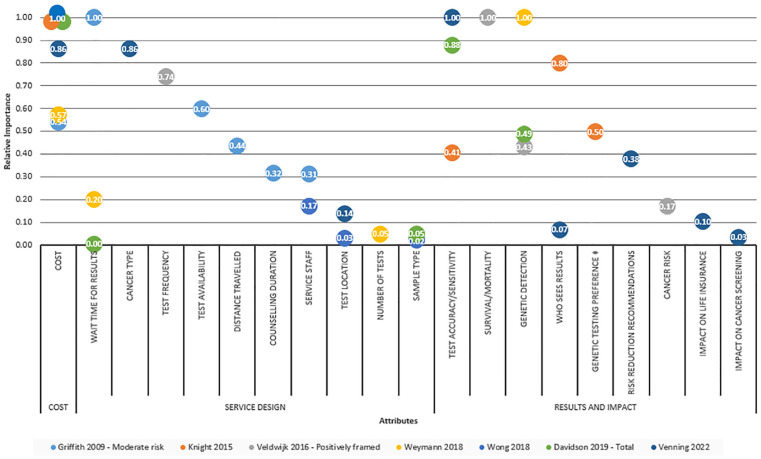
Relative importance of attributes. Values reflect the relative distance of all attributes to the most important attribute on a scale from 0 to 1 (1 indicating the most important attribute). Where multiple subgroups were reported, only 1 was selected for Figure 2 based on sample representation and positive framing (less emotional response). ^‡^Genetic testing preference was not included as an attribute in the discrete choice experiment but was calculated by comparing the utility associated with no test to the alternative of the average test.

Five studies reported attribute importance weight or rank.^24–27,29^ To facilitate between-study comparison, the relative importance of attributes was calculated in the remaining studies as the maximum difference in attribute level utility rescaled so the largest is 1.^23,28^ Relative attribute importance is displayed in Figure 2, which reflects the relative distance of all attributes to the most important attribute on a scale of 0 to 1, where 1 is the most important attribute.

Where attributes for cost, service design, and results/impact were all included, either cost or attributes related to the test result (accuracy, survival, detection) were considered most important by participants. Cost was considered most (or next to most) important in 5 studies^24–26,28,29^ but conversely of middling or least importance, depending on patient risk level, in another.^23^ Likewise, test accuracy, being the chance of a correct (or incorrect) test result, was considered least important in 1 study^25^ but of greatest importance in another.^29^ Regarding results and impact, participants were overall less concerned about attributes related to impact (resulting cancer risk, impact on life insurance, impact on future cancer screening) than those related to direct test results (detection, accuracy, survival). One study considered only cost and service design attributes,^23^ identifying wait time for results to be the most important attribute; otherwise, where results/impact attributes were also evaluated, wait time was deemed of low relative importance in the included studies.^24,26^ The least preferred attributes for service design were observed as test location,^28,29^ number of tests,^24^ and sample type.^26,28^

Four studies observed effects across different subgroups as illustrated in Appendix C. Veldwijk et al.^27^ considered the effect of negative (mortality) versus positive (survival) framing of the effectiveness outcome. A similar trend in preference was observed across both types of framing; however, survival was more important than colonoscopy frequency under positive framing, whereas colonoscopy frequency was more important than mortality in negative framing. Little variation in relative preference was observed by Davidson et al.^26^ when comparing multigene testing to nonmultigene or no testing; however, multigene testing did place greater importance on test sensitivity for mutations and probability of detecting uncertain variants. Griffith et al.^23^ found that the relative importance of attributes varied considerably when comparing across different risk groups. All groups valued wait time for results as most important. Meanwhile, the least important attributes varied across risk groups: test availability (high risk), counseling staff (moderate risk), and cost (low risk). Finally, Venning et al.^29^ considered relative importance across 3 latent classes. As expected, the “more is better” group placed most value on the cancer type attribute, “aim for accuracy” considered test accuracy to be most important, and “choose cheaper” respondents most valued the test cost attribute.^29^

#### Monetary value

Five studies calculated patient WTP for genetic testing, as presented in Table 4.^24–26,28,29^ These were in some cases described as WTP, although the naming was inconsistent and 3 different methods were used. Marginal WTP (mWTP) is obtained as the marginal rate of substitution between a single nonmonetary attribute and monetary attribute (cost) on the utility scale (i.e., it is given by the ratio of part-worth utilities). Money-equivalent values (MEV) are also obtained by translating utility differences into monetary terms by using the marginal utility of cost, but these utility differences result from differences across multiple attributes and give monetary values to comparisons of alternatives. Both mWTP and MEV can also be estimated by fitting the choice model in WTP space, which can lead to more reasonable distributions of mWTP when preference heterogeneity is included in the model.^32^ Compensating variation is a measure of WTP that applies when the choice set is changed (due to entry or exit of choices or changes in the quality or price of existing choices); the compensating variation is the amount of money that can be removed from an individual following a change in the choice set to return them to their utility level prior to the change.^33^ Of the included studies, 1 calculated WTP using compensating variation,^24^ while 4 calculated mWTP,^25,26,28,29^ of which 1 also calculated MEV.^25^

**Table 4:** Monetary Values (US$)

Service design	mWTP (95% CI)
Wong 2018	
Nonspecialist doctor v. nurse educator	−$21.57 (−25.03 to −18.11)
Public primary-care clinic v. hospital	−$7.65 (−11.66 to −3.64)
Buccal swab v. dried blood spot	$3.46 (1.03 to 5.88)
Private family clinic v. hospital	$10.05 (5.51 to 14.57)
Specialist doctor v. nurse educator	$37.04 (33.08 to 41.00)
Venning 2022	
Test for: bowel cancer v. pancreatic cancer	$15.17
GP v. online	$18.21
Test for: breast cancer v. pancreatic cancer	$24.65
Test for: prostate cancer v. pancreatic cancer	$31.16
Test for: multiple cancers in same test v. pancreatic cancer	$103.03
Overall Test	
Results and impact	MEV (95% CI)
Knight 2015	
Who sees results: primary care doctor v. genetics health professionals	$138.01 (22.38 to 258.61)
Chance of false negative: 0% v. 10%	$308.34 (200.17 to 420.24)
Chance of false negative: 10% v. 20%	$320.78 (203.90 to 442.62)
Chance of false negative: 0% v. 20%	$630.36 (471.22 to 795.73)
Who sees results: genetics health professionals v. insurance companies	$1,104.07 (893.95 to 1,321.65)
Who sees results: orimary care doctor v. insurance companies	$1,242.08 (1,013.31 to 1,483.28)
Davidson 2019	
Probability of VUS result: 20% v. 5%	$228.42 (141.82 to 333.43)
Probability of detecting deleterious mutation: 88% v. 80%	$271.73 (180.79 to 378.90)
Probability of VUS result: 40% v. 20%	$304.20 (188.37 to 444.94)
Probability of VUS result: 40% v. 5%	$532.63 (330.19 to 777.29)
Probability of detecting deleterious mutation: 80% v. 60%	$678.78 (450.35 to 947.25)
Probability of detecting deleterious mutation: 88% v. 60%	$949.42 (631.14 to 1,327.24)
	mWTP
Venning 2022	
No impact on life insurance eligibility or premiums v. impact	$11.92
Risk can be reduced through screening v. no available preventative measures	$26.08
Risk can be reduced by making lifestyle changes v. no available preventative measures	$26.29
Risk can be reduced through screening and lifestyle changes v. no available preventative measure	$43.85
Risk can be reduced by medication v. no available preventative measure	$49.79
Accuracy: 70% v. 60%	$51.28
Accuracy: 90% v. 60%	$125.11
Overall Test	MEV (95% CI)
Knight 2015	
Test v. no test^a^	$773.35 (591.82 to 967.31)
	WTP_cv_ (95% CI)
Weymann 2018	
MPS test 1 (60% detection rate, 3-wk wait for results) v. traditional diagnostic testing	$451.21 (338.41 to 564.02)
MPS test 2 (80% detection rate, 3-wk wait for results) v. traditional diagnostic testing	$1,404.40 (1,158.49 to 1649.18)
MPS test 3 (90% detection rate, 1.5-mo wait for results) v. traditional diagnostic testing	$1,738.30 (1,380.71 to 2,097.01)

All costs inflated to 2022 US$ rates using International Monetary Fund purchasing power parity values. Prices are inflated from the year of data collection or, where not reported, inflated from the year prior to publication. “v.” indicates “relative to,” that is, the testing alternative v. comparator or “change to” v. “change from.” CI, confidence interval; GP, general practitioner; MEV, money-equivalent values representing the additional amount of money that subjects would pay for tests with more preferred features; MPS, massively parallel sequencing; mWTP, marginal willingness to pay; VUS, variant of uncertain significance; WTP, willingness to pay; WTP_CV_, willingness to pay calculated using compensating variation.

a Test characteristics set to the mean values in the experimental design. Addition of the specified MPS test to an existing choice set of traditional diagnostic testing (40% detection rate, 3-mo wait for results) and no testing.

Regarding service design, participants most valued a test for multiple cancers compared with a test for a single cancer ($103.03)^29^ or a specialist doctor compared with a nurse educator ($37.04).^28^ The results and impact of genetic testing suggest participants place the highest value on a large improvement in probability of detecting deleterious mutation from 60% to 88% ($949.42)^26^ and on health professionals seeing the results rather than insurance companies ($1,242.08 and $1,104.07).^25^ Knight et al.^25^ suggested who sees the results, being health professionals rather than insurance providers, is of greater monetary value to participants than test accuracy is. Conversely, however, Venning et al.^29^ concluded that greater value is associated with increased accuracy, while the smallest value concerns the impact on life insurance eligibility or premiums.

Where studies consider mWTP for both service design and results/impact,^25,29^ attributes affecting results/impact hold the greatest monetary value, being whether the insurance company saw the test results^25^ or the test accuracy increasing from 60% to 90%.^29^ Values are not, however, consistently lower for service design attributes across the studies, as, for example, participants also place a high monetary value on the decision to test and whether to test for multiple cancers.

One study considered test compared with no test using MEV, yielding a value of $773.35.^25^ Only 1 study used compensating variation to estimate WTP, which it did by modeling the impact of introducing MPS testing in 3 scenarios to an existing choice set of traditional testing and no testing.^24^ These analyses revealed that participants were willing to pay for better detection rate (results) even with longer wait time for results (service design).^24^ A greater increase in WTP was observed between 60% and 80% detection rate than between 80% and 90%; however, the latter could be confounded by the increased wait time for results from 3 wk to 1.5 mo.^24^

An additional article by Griffith et al.^23^ did not calculate monetary values but observed that when an alternative with a cost of $6,570 (3,000 GBP in 2002) was presented, it was sometimes selected, indicating some participants were willing to pay this much (or more) sometimes. This study did not include an opt-out option, and the alternatives presented always incurred a cost of at least $3,285, with 1 of the alternatives (a constant comparator) always having a cost of $5,475. These methodological choices likely render any WTP estimates from the study unfit for purpose.

#### Predicted uptake of testing

Three studies predicted uptake of testing, as presented in Table 5.^24,25,28^ The highest predicted uptake was for a scenario for a hereditary colorectal cancer test in which the chance of a false-negative test result was 0%, cost of testing was $621.66 ($500 in 2015) and test results were shared with a primary care doctor. In this scenario, the predicted uptake of testing was 97% (95% confidence interval: 95−99),^25^ as determined by a probability-based sample of adults presented with a hypothetical level of colorectal cancer risk provided for context. In comparison, the least preferred scenario had an uptake of 3.7%, describing a “realistic base case” of buccal swab type, in a hospital, with a specialist doctor, costing $228.33 (S$175 in 2018), while the pricier option costing $391.42 (S$300 in 2018) had 0.0% uptake.^28^ This test was a single-nucleotide polymorphism gene test for breast cancer with preferences determined by women without history of breast cancer. For both tests, the cost would be incurred by the patient.

**Table 5:** Predicted Uptake of Testing

Knight 2015	% (95% CI)
Scenario 1 Cost of testing $621.66, shared with primary care doctor, no chance of FN	Scenario 1 = 97% (95, 99)
Scenario 2 Cost of testing $621.66, shared with insurance companies, 20% chance of FN	Scenario 2 = 41% (25, 57)
Weymann 2018	% (95% CI)
Genetic testing scenario 1 Detection rate (TP) = 60%, 1 test required, 3-wk wait for results	MPS scenario 1 = 34% (29, 39) Traditional diagnostic testing = 5% (2, 7)
Genetic testing scenario 2 Detection rate (TP) = 80%, 1 test, 3-wk wait for results	MPS scenario 2 = 73% (68, 79) Traditional diagnostic testing = 2% (0, 3)
Genetic testing, scenario 3 Detection rate (TP) = 90%, 1 test, 1.5-mo wait for results	MPS scenario 3 = 80% (74, 87) Traditional diagnostic testing = 1% (0, 3)
Wong 2018	%
Realistic base case Buccal swab, specialist doctor, hospital, $228.33	Realistic base case = 3.7%
Feasible alternative Buccal swab, nurse educator, hospital, $65.24	Feasible alternative = 60.5%
Cheaper alternative Buccal swab, specialist doctor, hospital, $65.24	Cheaper alternative = 87.0%
Pricier alternative Buccal swab, specialist doctor, hospital, $391.42	Pricier alternative = 0.0%
Most preferred alternative Buccal swab, specialist doctor, private family clinic, $65.24	Most preferred alternative = 88.9%

All costs inflated to 2022 US$ rates using International Monetary Fund purchasing power parity values. Prices are inflated from the year of data collection or, where not reported, inflated from the year prior to publication. FN, false negative; TP, true positive.

## Discussion

This review summarizes the relative importance and WTP for different attributes in genetic testing for hereditary cancer as identified across 7 included studies. Test effectiveness and detection rates were consistently important to respondents. Accuracy and cost, while also important, showed variation between studies. Studies also revealed patients and the public would be willing to pay for improved detection and clinician over insurance provider involvement.

### Conducting a DCE

#### Attribute selection

Attribute selection is key for DCE design. Several previous studies have highlighted a lack of rigor in the process and emphasized how this can introduce bias,^34^ which can be minimized by a multistep attribute-development process.^35^ Most studies included in this review used multiple stages to select attributes; however, only 1 reported the attribute selection process extensively enough to allow replication.^25^ Despite similar selection methods, the included attributes varied across studies. This may have been due to differing opinions expressed by respondents in focus groups or perhaps the different study aims and cancers targeted. For improved rigor and consistency, researchers conducting future DCEs in genetics should strive to follow the recommended 4-stage approach by Helter and Boehler^35^: raw data collection, data reduction, removing inappropriate attributes, and wording.

#### Study design

Included studies highlighted a number of design limitations. Five studies investigated only main effects,^23–27^ meaning potential interactions between attribute levels were not acknowledged, potentially confounding parameter estimates.^36^ Second, a forced-choice design was implied in more than half the studies,^26–29^ which, while beneficial in reducing missing data, could challenge external validity, overestimate preferences, and distort welfare measures.^36,37^ With regard dot genetic testing, opt-out or indifference options better reflect a real-world scenario in which an individual may choose not to test at all. Study design was also limited by lack of reporting on whether the variance covariance matrix was blocked diagonal, meaning identification was not checked, and parameter estimates may be confounded.^17^ In addition, piloting was poorly reported, if at all, and 1 study introduced design inefficiency by including a constant in each choice set, which discards information regarding the choice between attributes.^23^ Furthermore, we found an additional study reporting DCE design methods in genomic medicine for familial colorectal cancer; however, this was not included as no DCE results were reported.^38^

### Relative Importance of Attributes

Most studies included attributes for cost, service design, and results/impact. These studies considered a clinical attribute or measure of test effectiveness, such as detection or survival, to be of great importance in improving test utility.^24,26,27,29^ This is consistent with existing research in cancer treatment DCEs, in which treatment outcomes are considered most important to patients.^14^ In addition, preferences for higher detection rates and diagnostic yields can be seen in DCEs on genetic testing in a number of other populations and diseases.^39–41^

Meanwhile, in this review, accuracy was highly important in 1 study^29^ but of least importance in another.^25^ One factor influencing this discrepancy could be that while Venning et al.^29^ described the chance of a true-positive result, Knight et al.^25^ considered accuracy in terms of the risk of false negatives. Similar to attribute framing, which has been seen to influence WTP estimates in previous DCEs on colorectal cancer screening,^42^ this different focus may influence participant perception of accuracy. In addition, preferences could be driven by different health care systems, with the study by Knight et al. conducted in the United States and that by Venning et al. in Australia. The differing relative importance could also be a construct of the other attributes included in the DCE. For example, Venning et al. included twice as many attributes, including aspects such as cancer type, risk reduction measures, and testing process, which may alter relative attribute importance. Particularly of note, Venning et al. evaluated the polygenic risk score and found cancer type to be a highly valued attribute, whereas Knight et al. considered colorectal cancer alone, thus suggesting the general population may place higher value on evaluating a wider range of cancer types.

Inconsistences were also observed regarding the cost attribute. Studies evaluating all 3 aforementioned attribute categories ranked cost as most, or next to most, important,^24–26,28,29^ a finding consistent with genetic testing DCEs in other areas.^39–41^ Meanwhile, cost was of middle to low importance in the study by Griffith et al.,^23^ who considered only cost and service design attributes. There are multiple factors, besides co-occurring attributes, that may particularly influence the cost attribute. First, Griffith et al. conducted the DCE in Wales, where most health care is paid for by the NHS, making the concept, and therefore attribute, of paying for tests more hypothetical. Meanwhile the studies by Knight, Weymann, and Davidson were all conducted in the United States, where considerable fees can be associated with health care provision, even with a form of insurance, and Venning et al.^29^ reported the general public in Australia to have concerns around the impact of genetic testing on life insurance. Second, there could be cross-study differences in the financial status of the population and resulting ability to pay for testing. Third, as indicated in Griffith et al.,^23^ patient risk for developing cancer could be influential, with higher risk increasing the relative importance of service cost. Overall, however, this review suggests that participants in studies were sensitive to price; thus, lowering the cost of testing may lead to increased uptake.

Additionally of note, Griffith et al.^23^ reported that the wait time for test results had the greatest impact on the utility of testing services across all risk groups evaluated. This was contrary to the findings in 2 other studies,^24,26^ in which turnaround time was identified as of middle or low importance relative to other attributes. Griffith’s conclusion could perhaps be a construct of the other attributes, none of which consider clinical effectiveness of the test through, for example, detection rate or test sensitivity. In addition, Griffith et al. not only considered test characteristics but included attributes related to genetic counseling, such as distance and duration, which are not covered by other studies and may influence preferences.

The impacts of framing,^27^ genetic coverage,^26^ and risk level^23^ were explored through subgroup analysis. Veldwijk et al.^27^ investigated the impact of framing the effectiveness attribute positively (survival) or negatively (mortality). Positive framing concluded effectiveness as the most important attribute, while negative framing suggested it was colonoscopy frequency.^27^ These findings emphasize the importance of effectively communicating risk to patients to facilitate informed decision making, which has been widely discussed in the literature.^43,44^ Davidson, who considered genetic coverage, found little change in relative attribute importance; however, as would be expected, multigene testing valued test sensitivity for mutations and probability of detecting uncertain variants more than the nonmultigene or no-test group.^26^

### Monetary Value

Respondents were willing to pay for a test compared with no test at all^25^ and for a test for multiple cancers compared with a test for a single cancer.^29^ They also valued having a specialist doctor over less-specialized professionals^28^ and a private family clinic over a hospital,^28^ although the monetary value was smaller. Considering the results and impact of the test, participants placed the highest value on a large improvement in mutation detection (from 60% to 88%)^26^ and on health professionals rather than insurance companies seeing the test results.^25^ In addition, patients preferred traveling a greater distance to counseling, which authors suggest may be related to anonymity.^23^

Relative WTP regarding accuracy and the involvement or impact on insurance remains unclear. Participants in one study placed greater monetary value on who sees the results (health professionals v. insurance providers) than on improved accuracy,^25^ while respondents in another study conversely placed greater value on increased accuracy than on the resulting impact on life insurance.^29^ These are all aspects that should be carefully considered by policy makers and clinicians in designing or implementing a genetic test for hereditary cancer.

Challenges also arise in making cross-study WTP comparisons from heterogeneity in attributes, levels, and analysis. WTP estimates evaluated either only a select number of attribute levels, with levels differing between studies,^25,26,28,29^ or a combination of attributes to form a care program,^24,25^ both of which prevented insightful comparison or a conclusion of dominant preference. Furthermore, only 1 of the included studies used the Hicksian compensating variation method, which better reflects the choice nature of the task in calculating WTP.^24^ Hicksian compensating variation provides a common metric that could facilitate cross-study comparison of relative attribute importance.^45^ Overall, if the same method for assessing monetary value had been used in all studies, further WTP conclusions may have been possible.

WTP estimates did not appear to be adjusted to reflect ability to pay, limiting the interpretation of the results. While income level, which could provide context, was reported in some studies,^24,25,28^ the method of reporting was inconsistent. In addition, probability analysis was conducted in only 1 monetary valuation study,^28^ making it impossible to ascertain the impact of the attributes studied, or of sociodemographic variables, on respondents’ WTP. Lack of probability analyses hindered any opportunity to assess the impact of the attributes or sociodemographic characteristics on the predicted uptake of testing.

### Strengths and Limitations

While including conjoint studies may have provided a greater depth of evidence, this review was restricted to DCEs to ensure greater comparability. It is also consistent with existing research, which found very few conjoint analyses to add.^18^ Furthermore, this study focused only on genetic testing for hereditary cancer syndromes and polygenic cancer risk, providing greater relevance and utility to the population under consideration. Including predictive genetic testing for other complex diseases with a strong hereditary component may have increased the evidence base but come at the expense of precision given the differing nature and impact on preferences and quality of life related to different diseases. While the studies included some similar attributes, they were defined differently and contained different levels giving rise to heterogeneity, a limitation noted by other reviews in the area.^18^

### Future Research

Evaluation of the impact of sociodemographic characteristics on preferences for testing is limited, and existing research has reported contradictory results regarding willingness to undergo testing across sociodemographic groups.^11^ While several studies have reported an association between factors such as educational level and socioeconomic status on willingness to undergo testing, other studies have reported no association between these variables.^46–49^ Future studies should endeavor to include sociodemographic covariates and other respondent characteristics within the main model to assess the impact of factors related to the individual on willingness to undergo testing. This would help contextualize the relative importance of attributes across studies and assist in providing ability-to-pay context for WTP estimates.

Attention should also be paid to lesser studied attributes such as insurance provider interest and involvement, particularly in countries without free health care or social health insurance. Understanding the tradeoffs between various attributes, such as effectiveness and cost, across different health systems with different priorities would be particularly informative to policy makers and health service providers. Findings could help maximize the utility of testing services by, for example, understanding how changes in test or service characteristics may compensate for poorer clinical outcomes or higher costs, which at present seem most important to patients and the public.

## Conclusion

This review provides insight into the content and relative importance of attributes in genetic testing for hereditary cancer. In general, test result/impact is consistently important to both patients and the public; however, the impact of test accuracy needs further research to determine its relative impact. There is variation in the relative importance of cost, which we suggest could be dependent on health system, service cost, ability to pay, and cancer risk. Overall, participants were sensitive to prices; thus, lowering the cost of testing may lead to increased uptake. Finally, this review compared findings on WTP, which suggested individuals would be most willing to pay for a test that improves detection rates, identifies multiple cancers, and for which the results are shared with a doctor rather than with an insurance provider. Hence, these factors should be carefully considered by clinicians, researchers, and policy makers when attempting to assess the tradeoffs of genetic testing.

## Supplemental Material

sj-docx-1-mdm-10.1177_0272989X241227425 – Supplemental material for Preferences for Genetic Testing to Predict the Risk of Developing Hereditary Cancer: A Systematic Review of Discrete Choice Experiments Supplemental material, sj-docx-1-mdm-10.1177_0272989X241227425 for Preferences for Genetic Testing to Predict the Risk of Developing Hereditary Cancer: A Systematic Review of Discrete Choice Experiments by N. Morrish, T. Snowsill, S. Dodman and A. Medina-Lara in Medical Decision Making

## References

[1] Sullivan M. The new subjective medicine: taking the patient’s point of view on health care and health. Soc Sci Med. 2003;56(7):1595–604. 10.1016/s0277-9536(02)00159-4 12614708

[2] Ostermann J Brown DS de Bekker-Grob EW Mühlbacher AC Reed SD. Preferences for health interventions: improving uptake, adherence, and efficiency. Patient. 2017;10(4):511–4. 10.1007/s40271-017-0251-y PMC5534371 28597375

[3] Ghanouni A Smith SG Halligan S , et al. Public preferences for colorectal cancer screening tests: a review of conjoint analysis studies. Expert Rev Med Devices. 2013;10(4):489–99. 10.1586/17434440.2013.811867 23895076

[4] Rahner N Steinke V. Hereditary cancer syndromes. Dtsch Arztebl Int. 2008;105(41):706–14. 10.3238/arztebl.2008.0706 PMC2696972 19623293

[5] Miki Y Swensen J Shattuck-Eidens D , et al. A strong candidate for the breast and ovarian cancer susceptibility gene BRCA1. Science. 1994;266:66–71. 7545954 10.1126/science.7545954

[6] Wooster R Neuhausen SL Mangion J , et al. Localization of a breast cancer susceptibility gene, BRCA2, to chromosome 13q12-13. Science. 1994;265(5181):2088–90. 10.1126/science.8091231 8091231

[7] Lynch HT de la Chapelle A. Hereditary colorectal cancer. N Engl J Med. 2003;348(10):919–32. 10.1056/NEJMra012242 12621137

[8] Scott RJ. Familial Adenomatous Polyposis (FAP) and other polyposis syndromes. Hered Cancer Clin Pract. 2003;1(1):19–30.

[9] Vasen HF Blanco I Aktan-Collan K , et al. Revised guidelines for the clinical management of Lynch syndrome (HNPCC): recommendations by a group of European experts. Gut. 2013;62(6):812–23. 10.1136/gutjnl-2012-304356 PMC3647358 23408351

[10] Paluch-Shimon S Cardoso F Sessa C , et al. Prevention and screening in BRCA mutation carriers and other breast/ovarian hereditary cancer syndromes: ESMO Clinical Practice Guidelines for cancer prevention and screening. Ann Oncol. 2016;27(suppl 5):v103-10. 10.1093/annonc/mdw327 27664246

[11] Willis AM Smith SK Meiser B Ballinger ML Thomas DM Young MA. Sociodemographic, psychosocial and clinical factors associated with uptake of genetic counselling for hereditary cancer: a systematic review. Clin Genet. 2017;92(2):121–33. 10.1111/cge.12868 27643459

[12] Balmaña J Stoffel EM Emmons KM Garber JE Syngal S. Comparison of motivations and concerns for genetic testing in hereditary colorectal and breast cancer syndromes. J Med Genet. 2004;41(4):e44. 10.1136/jmg.2003.012526 PMC1735738 15060120

[13] Keogh LA Niven H Rutstein A Flander L Gaff C Jenkins M. Choosing not to undergo predictive genetic testing for hereditary colorectal cancer syndromes: expanding our understanding of decliners and declining. J Behav Med. 2017;40(4):583–94. 10.1007/s10865-016-9820-0 PMC6057776 28197815

[14] Bien DR Danner M Vennedey V Civello D Evers SM Hiligsmann M. Patients’ preferences for outcome, process and cost attributes in cancer treatment: a systematic review of discrete choice experiments. Patient. 2017;10(5):553–65. 10.1007/s40271-017-0235-y PMC5605613 28364387

[15] de Bekker-Grob EW Ryan M Gerard K. Discrete choice experiments in health economics: a review of the literature. Health Econ. 2012;21(2):145–72. 10.1002/hec.1697 22223558

[16] Hall R Medina-Lara A Hamilton W Spencer AE. Attributes used for cancer screening discrete choice experiments: a systematic review. Patient. 2022;15(3):269–85. 10.1007/s40271-021-00559-3 34671946

[17] Lancsar E Louviere J. Conducting discrete choice experiments to inform healthcare decision making: a user’s guide. Pharmacoeconomics. 2008;26(8):661–77. 10.2165/00019053-200826080-00004 18620460

[18] Ozdemir S Lee JJ Chaudhry I Ocampo RRQ . A systematic review of discrete choice experiments and conjoint analysis on genetic testing. Patient. 2022;15(1):39–54. 34085205 10.1007/s40271-021-00531-1

[19] Moher D Liberati A Tetzlaff J Altman DG ; PRISMA Group. Preferred reporting items for systematic reviews and meta-analyses: the PRISMA statement. PLoS Med. 2009;6(7):e1000097. 10.1371/journal.pmed.1000097 PMC2707599 19621072

[20] Kilambi V Johnson FR González JM Mohamed AF. Valuations of genetic test information for treatable conditions: the case of colorectal cancer screening. Value Health. 2014;17(8):838–45. 10.1016/j.jval.2014.09.001 PMC4492688 25498779

[21] Veldwijk J Lambooij MS Kallenberg FG , et al. Preferences for genetic testing for colorectal cancer within a population-based screening program: a discrete choice experiment. Eur J Hum Genet. 2016;24(3):361–6. 10.1038/ejhg.2015.117 PMC4755369 26036860

[22] Veldwijk J Groothuis-Oudshoorn CGM Kihlbom U , et al. How psychological distance of a study sample in discrete choice experiments affects preference measurement: a colorectal cancer screening case study. Patient Prefer Adherence. 2019;13:273–82. 10.2147/PPA.S180994 PMC6388728 30863017

[23] Griffith GL Edwards RT Williams JM , et al. Patient preferences and National Health Service costs: a cost-consequences analysis of cancer genetic services. Fam Cancer. 2009;8(4):265–75. 10.1007/s10689-008-9217-5 18821034

[24] Weymann D Veenstra DL Jarvik GP Regier DA. Patient preferences for massively parallel sequencing genetic testing of colorectal cancer risk: a discrete choice experiment. Eur J Hum Genet. 2018;26(9):1257–65. 10.1038/s41431-018-0161-z PMC6117311 29802320

[25] Knight SJ Mohamed AF Marshall DA Ladabaum U Phillips KA Walsh JME . Value of genetic testing for hereditary colorectal cancer in a probability-based US online sample. Med Decis Making. 2015;35(6):734–44. 10.1177/0272989X14565820 PMC4501912 25589525

[26] Davidson BA Ehrisman J Reed SD Yang J-C Buchanan A Havrilesky LJ. Preferences of women with epithelial ovarian cancer for aspects of genetic testing. Gynecol Oncol Res Pract. 2019;6(1):1. 30693090 10.1186/s40661-019-0066-8 PMC6341581

[27] Veldwijk J Essers BA Lambooij MS Dirksen CD Smit HA de Wit GA. Survival or mortality: does risk attribute framing influence decision-making behavior in a discrete choice experiment? Value Health. 2016;19(2):202–9. 10.1016/j.jval.2015.11.004 27021754

[28] Wong XY Groothuis-Oudshoorn CG Tan CS , et al. Women’s preferences, willingness-to-pay, and predicted uptake for single-nucleotide polymorphism gene testing to guide personalized breast cancer screening strategies: a discrete choice experiment. Patient Prefer Adherence. 2018;12:1837–52. 10.2147/PPA.S171348 PMC6154732 30271127

[29] Venning B Saya S De Abreu Lourenco R Street DJ Emery JD. Preferences for a polygenic test to estimate cancer risk in a general Australian population. Genet Med. 2022;24(10):2144–54. 10.1016/j.gim.2022.07.011 35947108

[30] Ryan M Gerard K Amaya-Amaya M . The Economics of Non-market Goods and Resources. Bateman IJ , ed. Dordrecht (the Netherlands): Springer; 2008.

[31] Daly A Dekker T Hess S. Dummy coding vs effects coding for categorical variables: clarifications and extensions. J Choice Model. 2016;21:36–41.

[32] Train K Weeks M. Discrete choice models in preference space and willingness-to-pay space. In: Scarpa R Alberini A eds. Applications of Simulation Methods in Environmental and Resource Economics. Dordrecht (the Netherlands): Springer; 2005. p 1–16.

[33] Lancsar E Savage E. Deriving welfare measures from discrete choice experiments: inconsistency between current methods and random utility and welfare theory. Health Econ. 2004;13(9):901–7. 10.1002/hec.870 15362181

[34] Coast J Al-Janabi H Sutton EJ , et al. Using qualitative methods for attribute development for discrete choice experiments: issues and recommendations. Health Econ. 2012;21(6):730–41. 10.1002/hec.1739 21557381

[35] Helter TM Boehler CEH . Developing attributes for discrete choice experiments in health: a systematic literature review and case study of alcohol misuse interventions. J Subst Use. 2016;21(6):662–8. 10.3109/14659891.2015.1118563 PMC5022136 27695386

[36] Mandeville KL Lagarde M Hanson K. The use of discrete choice experiments to inform health workforce policy: a systematic review. BMC Health Serv Res. 2014;14:367. 25179422 10.1186/1472-6963-14-367 PMC4161911

[37] Veldwijk J Lambooij MS de Bekker-Grob EW Smit HA de Wit GA. The effect of including an opt-out option in discrete choice experiments. PLoS One. 2014;9(11):e111805. 10.1371/journal.pone.0111805 PMC4218820 25365169

[38] Bennette CS Trinidad SB Fullerton SM , et al. Return of incidental findings in genomic medicine: measuring what patients value-development of an instrument to measure preferences for information from next-generation testing (IMPRINT). Genet Med. 2013;15(11):873–81. 10.1038/gim.2013.63 PMC3823641 23722871

[39] Goranitis I Best S Christodoulou J Boughtwood T Stark Z. Preferences and values for rapid genomic testing in critically ill infants and children: a discrete choice experiment. Eur J Hum Genet. 2021;29(11):1645–53. 10.1038/s41431-021-00874-1 PMC8560904 33811253

[40] Buchanan J Blair E Thomson KL , et al. Do health professionals value genomic testing? A discrete choice experiment in inherited cardiovascular disease. Eur J Hum Genet. 2019;27(11):1639–48. 10.1038/s41431-019-0452-z PMC6870981 31186546

[41] Blumenschein P Lilley M Bakal JA Christian S. Evaluating stakeholder’s perspective on referred out genetic testing in Canada: a discrete choice experiment. Clin Genet. 2016;89(1):133–8. 10.1111/cge.12592 25827301

[42] Howard K Salkeld G. Does attribute framing in discrete choice experiments influence willingness to pay? Results from a discrete choice experiment in screening for colorectal cancer. Value Health. 2009;12(2):354–63. 10.1111/j.1524-4733.2008.00417.x 18657102

[43] Vass C Rigby D Payne K. “I was trying to do the maths”: exploring the impact of risk communication in discrete choice experiments. Patient. 2019;12(1):113–23. 10.1007/s40271-018-0326-4 30099692

[44] Pearce A Harrison M Watson V , et al. Respondent understanding in discrete choice experiments: a scoping review. Patient. 2021;14(1):17–53. 33141359 10.1007/s40271-020-00467-y PMC7794102

[45] Lancsar E Louviere J Flynn T. Several methods to investigate relative attribute impact in stated preference experiments. Soc Sci Med. 2007;64(8):1738–53. 10.1016/j.socscimed.2006.12.007 17257725

[46] Aktan-Collan K Mecklin JP Järvinen H , et al. Predictive genetic testing for hereditary non-polyposis colorectal cancer: uptake and long-term satisfaction. Int J Cancer. 2000;89(1):44–50. 10719730

[47] Bleiker E Wigbout G van Rens A Verhoef S Van’t Veer L Aaronson N. Withdrawal from genetic counselling for cancer. Hered Cancer Clin Pract. 2005;3(1):19–27. 20223026 10.1186/1897-4287-3-1-19 PMC2837064

[48] Anderson B McLosky J Wasilevich E Lyon-Callo S Duquette D Copeland G. Barriers and facilitators for utilization of genetic counseling and risk assessment services in young female breast cancer survivors. J Cancer Epidemiol. 2012;2012:298745. 23150731 10.1155/2012/298745 PMC3485517

[49] Culver J Burke W Yasui Y Durfy S Press N. Participation in breast cancer genetic counseling: the influence of educational level, ethnic background, and risk perception. J Genet Couns. 2001;10(3):215–31.

