# Supplementary material for: Preferences for Genetic Testing to Predict the Risk of Developing Hereditary Cancer: A Systematic Review of Discrete Choice Experiments
Source: Med Decis Making. 2024 Feb 7;44(3):252–68. doi: 10.1177/0272989X241227425 (PMC10988993; doi:10.1177/0272989X241227425)
Supplement: sj-docx-1-mdm-10.1177_0272989X241227425 – Supplemental material for Preferences for Genetic Testing to Predict the Risk of Developing Hereditary Cancer: A Systematic Review of Discrete Choice Experiments [file sj-docx-1-mdm-10.1177_0272989X241227425.docx]

Appendices

# Appendix A: Search terms used to identify studies related to genetic testing in oncology

Discrete choice studies were identified using pre-tested published search terms from de Bekker-Grob et al. 2012. (15). These terms included: ‘discrete choice experiment(s)’, ‘discrete choice model(l)ing’, ‘stated preference’, ‘part-worth utilities’, ‘functional measurement’, ‘paired comparisons’, ‘pairwise choices’, ‘conjoint analysis’, ‘conjoint measurement’, ‘conjoint studies’, ‘conjoint choice experiment(s)’. All hits for discrete choice studies were then downloaded into an EndNote library to create a database.

This Endnote database of discrete choice studies was then searched to identify studies related to genetic testing in oncology. Titles and abstracts were searched for the following terms for adult hereditary cancer syndromes combined with terms for malignancy and genetic testing:

1. lynch OR HNPCC OR BRCA OR hereditary breast ovarian cancer OR HBOC OR Li-Fraumeni OR FAP OR familial adenomatous polyposis
2. cancer OR carcinoma OR tumour OR tumor OR metastasis OR metastases OR oncology OR neoplasm OR malignancy
3. gene OR genetic OR genome OR DNA OR genotype OR hereditary
4. 2 AND 3
5. 1 OR 4

# Appendix B: Quality appraisal

|  | Question | Griffith 2009 | Knight 2015 | Veldwijk 2016 | Weymann 2018 | Wong 2018 | Davidson 2019 | Venning 2022 |
| --- | --- | --- | --- | --- | --- | --- | --- | --- |
| **Conceptualizing the choice process** | Choice, ranking or rating test? | Choice | Choice | Choice | Choice | Choice | Choice | Choice and ranking |
|  | Binary response, pairs, or multiple options? | Pairs | Pairs | Pairs | Pairs | Pairs | Pairs | Pairs |
|  | Generic or labelled? | Generic | Generic | Generic | Generic | Generic | Generic | Generic |
|  | Opt-out, neither, status quo options? | Indifference | Opt-out | Opt-out; forced choice followed by opt out. However, opt out option was not modelled. | Neither | No | No | No |
|  | Justification for above? | None provided | External validity and to facilitate uptake predictions | Inappropriate to set all attribute-levels as zero in the opt-out alternative given the inclusion of clinical variables | External validity | None provided | None provided | None provided |
|  | Was the task incentive compatible? | Yes | Yes | Yes | Yes | Yes | Yes | Yes |
| **Attribute selection** | How were attributes derived and validated? | Four levels based on a previous study, 2 to facilitate evaluation of study aims | Literature review and focus groups with clinical experts, at risk-patients and population-risk patients | Literature, expert interviews, interviews with target population | Literature review, consultation with experts, focus group and cognitive interviews with patients | Focus groups with screening-age women, existing literature, clinicians and health service researchers | Literature review and consultation with genetic testing counsellors | Literature review, expert opinion, consumer focus groups |
|  | Was the number of attributes appropriate? | Yes (n=6) | Yes (n=4) | Yes (n=4) | Yes (n=4) | Yes (n=4) | Yes (n=5) | Yes (n=8) |
|  | Was the coverage appropriate? | Included test and service attributes | Included test attributes | Included clinical attributes | Included test attributes | Included test attributes | Included test and clinical attributes | Included test and clinical attributes |
|  | What form was used: generic or alternative specific? | Alternative specific | Alternative specific | Alternative specific | Alternative specific | Generic | Generic | Generic |
|  | Was price included? | Yes | Yes | No | Yes | Yes | Yes | Yes |
|  | Was risk included? | No | Risk status was incorporated within the DCE questionnaire, each choice set had the same risk level across both alternatives. However risk was not analysed as an attribute. The risk varied across different choice sets. | Yes. Risk of colorectal cancer | No | No | No. Though did include chance of finding mutation if have one, or of finding an uncertain variant | No. Though did include attribute for risk reduction measures |
| **Levels** | How were they derived and validated? | Not reported | Focus group discussions and previous literature. | Representative of the 3 most common familial cancers | Levels representative of MPS or traditional diagnostic testing | Focus groups with screening-age women, existing literature, clinicians and health service researchers | Not reported | Literature review, expert opinion, consumer focus groups |
|  | Was the number of levels per attribute appropriate? | Yes | Yes | Yes | Yes | Yes | Yes | Yes |
|  | Was an appropriate range used | Yes | Yes | Yes | Yes | Yes | Yes | Yes |
|  | Were the levels evenly spaced? | Yes | No | No | No | Yes | No | Yes, where possible |
| **Experimental design** | What type of design was used? Full factorial or fractional factorial? | Fractional factorial design (23 choice sets) | Fractional factorial design (36 choice sets) | Fractional factorial design (9 choice sets) | Fractional factorial design (36 choice sets) | Fractional factorial design (10 choice sets) | Fractional factorial design (10 choice sets) | Fractional factorial design (12 choice sets) |
|  | How were profiles generated and allocated to choice sets? | SPEED 2.1 software was used to generate a small fractional factorial design which consisted of 24 profiles. One profile was selected that described the current practice at the CGSW, this profile was used as a constant comparator against which all other profiles were compared in the choice sets. | Employed D-optimal algorithm generate a fractional factorial main effects experimental design in SAS version 9.2. | Ngene 1.0 (Choice metrics pty ltd, 2011, St leonards, NSW, Australia) software used to generate D-efficient design | Applied D-optimal procedures to generate a statistically efficient choice-based design using the SAS %ChoicEff macro | Sawtooth Lighthouse Studio 6.4.4. using balanced overlap method | Not reported | Orthogonal array with 108 runs using 4 generators. Partial profiles with 2 overlapping attributes used to improve complexity and efficiency |
|  | What are the properties of the design? | Main effects only | Main effects only | Main effects only | Main effects only | Main effects and interactions | Main effects only | Main effects and interactions for female/breast cancer and male/prostate cancer |
|  | What is the efficiency of the design? | Not reported | Not reported | Not reported | Not reported | Not reported | Not reported | Not reported |
|  | Was identification checked? (e.g. is the variance-co-variance matrix block diagonal?) | Not reported | Not reported | Not reported | Not reported | Not reported | Not reported | Not reported |
|  | Was the design blocked into versions? If so how were choice sets allocated to versions? Were the resulting properties of the versions checked? | n/a | Yes. Survey blocked into four versions. | n/a | Yes, blocked into two versions of the survey. Blocks were orthogonal to the attribute levels to ensure that parameter estimates were independent of each other | n/a | Yes. 21 blocks of ten 2-alternative choice sets | Yes. 36 blocks of 12 choice sets. Minimum of 20 respondents for each block |
|  | Were respondents randomly allocated to versions | n/a | Yes | n/a | Not reported | n/a | Not reported | Not reported |
|  | How many choice sets were considered per respondent? | 23 | 9 | 9 | 16 | 10 | 10 | 12 |
|  | If some profiles were implausible - how was implausibility defined and how was it addressed? | n/a | n/a | n/a | n/a | n/a | n/a | Participants allocated to cancer type based on gender |
| **Questionnaire design** | Was an appropriate level of background and contextual information provided? | Yes, Respondents received an information pack on genetic cancer. Attributes included in this DCE did not require definitions as easily interpreted. | Introduction to the topic of genetic testing for colorectal cancer and questions, and comprehension of risk information. Graphical representations and pictures were used to define concepts used in the choice tasks, such as risk, false-negative test result. | Yes. Detailed information regarding on the meaning of attributes and levels as well as an explanation on how to complete a choice task and provided an example question. | Yes. Questionnaires began with an educational component explaining the attributes and levels. | Yes. Respondents received a two-page explanation on SNP gene testing before completing the DCE | Yes. Respondents viewed a 7-min educational video on genetic testing, the attributes and associated levels | Yes. Survey included plain language statement and consent form including PRS test description, study attribute and level description, testing scenario vignette |
|  | Were the task instructions appropriate | Yes | Yes, participants instructed to indicate in which situation they would prefer to undergo testing given their hypothetical risk status presented within the DCE. | Yes, participants advised to assume that they have been referred for genetic testing due to clinical findings/ family history, and instructed to indicate in which situation they would choose to undergo testing given the hypothetical risk of carrying a high-risk variant presented within the DCE | Yes | Yes, part A asked to choose between two alternatives, part B asked if would really go for it in real life | Not reported | Yes, participants asked to imagine they are attending a GP check-up and are offered 2 different DNA tests for cancer risk. Asked ‘which DNA test would you choose?’ |
|  | Was the medium used to communicate attribute/level information (words, pictures or multimedia) appropriate? | Yes | Yes | Unclear, no example provided | Yes | Yes | Yes | Yes |
| **Piloting** | Was coverage of the attributes and levels checked? | No piloting of the DCE was reported | Yes. "participants saw the characteristics [attributes] and levels as relevant and of concern" | Yes. attribute-level estimates retrieved from pilot study served as input for the design of the final DCE. | No piloting of the DCE was reported | Not reported whether piloting considered coverage of attributes and levels | Not reported whether piloting considered coverage of attributes and levels | Not reported whether piloting considered coverage of attributes and levels |
|  | Was understanding and complexity checked? | No piloting of the DCE was reported | 10 respondents completed the DCE thinking and answered debriefing questions to check understanding. | 90 respondents completed the questionnaire to check understanding and complexity. 4 were observed completing the questionnaire thinking aloud | No piloting of the DCE was reported | 20 respondents completed pilot DCE. Responses led to question modification | First 4 subjects recruited used to pilot. Informal qualitative interviews used to assess ease of use and level of understanding | 105 respondents completed pilot DCE with post-survey evaluation of clarity of survey instructions, explanation of PRS testing, and choice task difficulty. Refinements made |
|  | Was the length and timing checked? | No piloting of the DCE was reported | Not reported, although it is stated that each survey was designed to take 20 mins, including additional data collection. | Not reported, respondents completed only 9 choice sets however other data was also collected so potential fatigue/ boredom effects | No piloting of the DCE was reported | Not reported | Not reported | Not reported |
| **Population** | Appropriate for the research question? | Individuals at risk of genetic cancer referred to the Cancer Genetics Service in Wales. | Representative probability-based sample of US population, aged 50+ | Representative sample of 55–65-year-old Dutch residents | Patients had a personal and/or family history of colon cancer and/or polyposis or other features of Lynch syndrome, who were referred to the University of Washington Genetic Medicine Clinic for Usual Care genetic testing for Mendelian CRC risk | Representative sample of Singaporean women aged 40-69 without history of breast cancer | Women age 18+ with non-mucinous EOC. | Australian participants aged 18+ with quotas for age and gender used to match general population |
| **Sample** | Were inclusion and exclusion criteria explicit | Not reported | Those that did not meet quality standards were excluded, and those with incomplete responses. | Those that had previously participated in CRC screening programme and those that completed the survey at an unlikely fast pace (within 10 minutes) were excluded | Patients with a history of genetic testing for colon cancer or polyps and those with a high probability that their condition was caused by one specific gene were excluded from this study. | Screening age women (age 40-69) selected based on pre-specified ethnic quotas with Singapore citizenship and no prior history of breast cancer | Women age 18+ with diagnosis of non-mucinous EOC referred for germline genetic testing. Included women with a family or personal history of cancer | Australian participants aged 18+ |
|  | Was sample size appropriate for model estimation | 115 patients. No sample size calculation reported | 451 participants. No sample size calculation reported. | 1045. No sample size calculation reported. | 122 participants. No sample size calculation reported. | 300. Minimum sample size of 75 based on Orme’s Rule of Thumb | 114 patients. No sample size calculation reported | 1002 participants. No sample size calculation reported |
| **Data collection** | What recruitment method was used? | Consecutive sampling via three Cancer Genetics Services and Wales | Recruitment via Knowledge Networks panel. | Recruited via an existing panel of the Dutch population | Recruited patients included in the Next Medicine study | Multistage cluster sampling by a market research company | Eligible participants identified | Recruited using an independent, actively managed online panel |
|  | How were data collected? | Mailed questionnaires | Online survey | Online survey | Online survey | Face-to-face survey | Not reported | Online survey |
|  | What was the response rate? | 54.50% | 70.00% | 79.10% | 66.00% | 50.20% | Not reported | 83.00% |
|  | Were incentives used to enhance response rates | No | No | No | No | Yes, S$30 shopping vouchers as compensation | No | Yes, paid through online panel membership on per-minute basis. Amount not reported |
| **Coding of data** | Was coding explicitly discussed? | Attribute levels in this analysis were effects coded. | Effects coding used in the model to estimate the reference levels as the sum of the rest of the preference weights for each attribute. | All attributes were effects coded to account for potential nonlinearity. Reference category coded as -1 and so the sum of effect coded attributes is zero. | Effects coding was used for the proportion of individuals with a variant detected. All other variables coded as continuous variables. | Out-of-pocket cost coded as continuous. All other attributes were effects-coded | Effect-coded variables | All attributes dummy coded across both models |
|  | Was the coding appropriate for effects to be estimated? | Yes | Yes | Yes | Yes | Yes | Yes | Yes |
| **Econometric analysis** | Were the estimation methods appropriate given the experimental design? | Multinomial logit regression on Limdep 7.0/Nlogit 2.0 | Random-parameters logit models using NLOGIT 4.0 | Panel mixed-logit model constructed using Nlogit 5.0 | Error-component mixed logit model | Mixed logit model using Stata 14.2 | Random-parameters logit regression in Stata 15 | Mixed logit models and latent class analysis using Stata 17 |
|  | Was the functional form of the indirect utility functions appropriate given the experimental design? | Model is limited by strict assumptions regarding identical distribution of errors and assumption of independence of irrelevant alternatives. | Appropriate in cases where there is considerable preference heterogeneity | Mixed logit model allows for preference heterogeneity and relaxes the independence of irrelevant alternative assumption. | Mixed logit model allows for preference heterogeneity and relaxes the independence of irrelevant alternative assumption. | Mixed logit accounts for preference heterogeneity around parameter estimates among respondents | Appropriate where considerable preference heterogeneity | Mixed logit accounts for panel nature of data and preference heterogeneity |
|  | Were alternative specific constants included? | No | ASC used for the opt-out alternative | No | ASC used for the opt out alternative | ASC used for choosing the left-sided alternative and not go for the test in real life | No | Not reported |
|  | Were sociodemographics and other covariates included? | No | No | No | No | Studied interactions between significant attribute levels and respondent characteristics | No | No |
|  | Was goodness of fit considered? | No, but did calculate McFadden’s R^2^ | No | Yes. Model fit tests (AIC, BIC, log likelihood) used to assess which model was most suitable for the data | No | No | No | No. Though did consider number of latent classes using Akaike and Bayesian information criteria |
| **Validity** | Was internal or external validity investigated? | Internal validity; dominance, transience and non-satiation tests were included in the questionnaire. Respondents that violate random utility theory axioms were not excluded. | Not reported | Not reported | Not reported | Not reported | Not reported | Not reported |
|  | Were answers for any respondent deleted and if so on what basis? | None deleted. | Those with incomplete answers and who choose the same alternative indiscriminately. | Those who completed at an unlikely speed were excluded. | None deleted. | None deleted | None deleted | None deleted |
| **Interpretation** | Was the interpretation appropriate given coding of data? | Yes | Yes | Yes | Yes | Yes | Yes | Yes |
|  | Were the results in line with a priori expectations? | Mostly. However greater utility associated with travelling 40 miles compared to 20; high-risk group preferred £2000 cost of testing than £1500 suggests assumptions based on price/quality. | Yes | Yes | Yes | Yes | Yes | Mostly. Results differ in finding medication more attractive than lifestyle modification or screening for risk reduction |
|  | Were relative attribute effects compared using a common and comparable metric? | No. Only attribute-level estimates were reported not compared using a comparable metric. | Importance weights and relative importance of attributes calculated by scaling all attributes estimates relative to the attribute that had the largest impact on utility. | Importance weights and relative importance of attributes calculated by scaling all attributes estimates relative to the attribute that had the largest impact on utility. | Importance weights and relative importance of attributes calculated by scaling all attributes estimates relative to the attribute that had the largest impact on utility. | Part-worth utilities or preference weights calculated for all attribute weights | Preference weights estimated and rescaled on a scale of 0 to 10. Importance weights calculated as fraction of 100 | Multicancer and specific cancer PRS tests compared relative to reference level pancreatic cancer |
| **Welfare and policy analyses** | Was willingness to pay estimated using welfare theoretic compensating variation? | No. WTP assessed by including a cost attribute | No. Study reports the WTP for genetic testing (assuming mean values for test characteristics) compared to no testing divided by cost | No | Yes. Study reported WTP for 3 testing scenarios compared to traditional diagnostic testing using compensating variation formula. | No. WTP calculated using marginal rates of substitution | No. Used cost attribute to calculate money-equivalent values | No. WTP estimated by taking the ratio of the coefficients for attribute levels over mean scaled coefficient for cost |
|  | Was probability analysis undertaken? | No | No | n/a | No | Assessed heterogeneity in marginal WTP by constructing post-estimation probability density and cumulative density functions across ethnicity | No | No |
|  | Were marginal rates of substitution calculated? | No | Study reported the marginal WTP for improvements in test attributes. | No | No | Preferences among ethnic groups compared using marginal rates of substitution | No | No |

# Appendix C: Relative importance of attributes across subgroups

Values reflect the relative distance of all attributes to the most important attribute on a scale from 0 to 1 (1 indicating the most important attribute).
